# Supplementary material for: Deep learning-based tumor microenvironment segmentation is predictive of tumor mutations and patient survival in non-small-cell lung cancer
Source: BMC Cancer. 2022 Sep 21;22:1001. doi: 10.1186/s12885-022-10081-w (PMC9490924; doi:10.1186/s12885-022-10081-w)
Supplement: Supplementary file 1 — Additional file 1: Supplementary material. [file 12885_2022_10081_MOESM1_ESM.docx]

**Supplementary Information: Deep learning based tumor microenvironment segmentation is predictive of tumor mutations and patient survival in non-small-cell lung cancer**

Alicja Rączkowska^1^, Iwona Paśnik^2^, Michał Kukiełka^1^, Marcin Nicoś^3^, Magdalena A. Budzinska^4^, Tomasz Kucharczyk^3^, Justyna Szumiło^2^, Paweł Krawczyk^3^, Nicola Crosetto^5,6^, Ewa Szczurek^1,*^

^1^Faculty of Mathematics, Informatics and Mechanics, University of Warsaw

^2^Department of Clinical Pathomorphology, Medical University of Lublin

^3^Department of Pneumology, Oncology and Allergology, Medical University of Lublin

^4^Ardigen

^5^Division of Genome Biology, Department of Medical Biochemistry and Biophysics, Karolinska Institutet

^6^Science for Life Laboratory

*Corresponding author

## **Abstract**

This supplementary information includes **Supplementary Methods**, **Supplementary Results**, **Supplementary Discussion**, **Supplementary Figures 1-6** and **Supplementary Tables 1-3**.

## **Supplementary Methods**

**Hematoxylin and eosin staining**

FFPE tissue was cut into 3 µm fragments and placed on glass slides. The slides were then stained with hematoxylin and eosin (H&E) in Leica Autostainer XL device (Leica Biosystems, USA), with pre-staining xylene and ethanol wash steps and 7 min of hematoxylin, and 20 sec of eosin. Post-staining wash steps were also included in the program. The glass slides were cover-slipped directly after the staining procedure. The H&E stained slides were scanned using the Aperio ScanScope CS2 device (Leica Biosystems, USA) equipped with an 20x Olympus microscope lens. The images were stored on an internal server in the form of SVS files, which were further analyzed.

**Dataset with treatment information included**

In addition to image-based TIP and TMEC features, clinical data and mutation status (see **Methods** in the main text), we also added treatment information to the data used for survival prediction analysis. All treatment data was extracted through the GDC REST API. Due to the fact that treatment information was not available for all 411 previously considered patients, we prepared a separate dataset containing 377 patients and the following binary features: targeted molecular therapy, chemotherapy, radiotherapy, combined chemo- and radiotherapy. We provide this dataset in **Additional File 3**.

## **Supplementary Results**

**Clinical features identified as significant for patient survival**

From among clinical features, statistically significant association was found for the advanced stage, locally advanced stage and age $\geq$ 65 features (Wald test *p-value* < 0.05). As expected, both for patients with locally advanced stage and with advanced stage the hazard was increased compared to early stage, and with the advanced stage having the largest estimated hazard ratio of the stages. Similarly, as expected, for older patients the hazard was larger. The estimated hazard ratio for light smokers was increased as compared to the non-smokers. Seemingly counter-intuitively, the hazard ratio for heavy smokers decreased. This may be explained by the fact that smoking is a well known risk factor for morbidity in lung cancer, but not mortality. In fact, patients who smoke after lung cancer diagnosis have a worse prognosis than patients who quit smoking. However, patients who smoke heavily before diagnosis may have a high tumor mutation burden, a high number of tumor antigens, and an active immune system with higher sensitivity to immune checkpoint inhibitors, which may prove beneficial for overall survival [(1)](https://www.zotero.org/google-docs/?2eHHgd). From among mutation features, *TP53* and *STK11* statistically significantly increased the death hazard (Wald test *p-value* < 0.05), which agrees with the results from the independent Kaplan-Meier analysis (**Sup. Fig. 2**).

## **Supplementary Discussion**

**Limitations of the proposed approach**

Our approach has several limitations. In the mutation classification tasks, we used simple machine learning models – logistic regression and random forest. An end-to-end deep learning model might give better results, however, as discussed above, these models suffer when it comes to interpretability. Another limitation is the fact that ARA-CNN works on a patch-based basis. An alternative to that is a cell-based classifier, which could produce more fine-grained segmentations and in turn enable a more precise computation of spatial statistics. On the other hand, with a patch-based approach, a suitably small patch size can be selected, as we did in this study. Such small patches can be assumed to be homogeneous when it comes to cell types and can enable a precise computation of summary statistics such as the TIP and TMEC features, that we have introduced here. However, due to the aggregation-based nature of these features, pathological events such as angioinvasions are hard to model properly. This is consistent with human-level perception of H&E images, as angioinvasions are hard to assess even for pathologists. Lastly, to apply our approach to another cancer type, one would need to retrain the ARA-CNN model, which necessitates substantial input from a trained pathologist. This training effort can be minimized by utilizing the active learning component of ARA, which shortens the number of iterations required to build an effective training dataset. For colorectal cancer, a pre-trained model is available from a previous study [(2)](https://www.zotero.org/google-docs/?6l8J3h).

Compared to data from antibody-based methods for multiplex cancer tissue imaging analyzed by several recent studies [(3–6)](https://www.zotero.org/google-docs/?23gG5J), the data analyzed in this work is limited in terms of the number of cell types and their states it allows to identify. In particular, many types of immune cells are not distinguishable in H&E slides even by expert pathologists. In contrast to multiplexed antibody imaging data, however, H&E slides are abundant and routinely used in the clinic and are becoming more commonly digitized. Thus, predictive models operating on H&E data are more likely to be adopted in clinical practice.

The computation of TIP and TMEC features was performed based on whole TCGA tissue slides, without selecting specific regions of interest (ROI). This may be viewed as a limitation of the TIP feature, as if the tumor area in a given slide is small, then its corresponding TIP component is underrepresented. As such, TIP depends on the selection of the analyzed region. Still, it is a valid summary statistic of the H&E data available for the patients, and whole-slide information was successfully used previously for various classification tasks, such as [(7)](https://www.zotero.org/google-docs/?wgkDvg). In contrast, the TMEC feature by definition quantifies the TME only within margins around the tumor areas. Thanks to the fact that we are able to identify the margins computationally, it is not necessary to manually annotate ROIs and the analysis pipeline can be largely automated.

The necessity to verify the results of ARA-CNN segmentations, as performed for the TCGA data, can be seen as another downside of the presented approach. Specifically, we manually screened the segmentation results of ARA-CNN and excluded slides which were found to be erroneous, either due to contamination by colored ink or due to wrong classification results provided by the model (it must be noted that overall the latter event was very rare: 21 out of 506 slides, or 4%). On the one hand, the model cannot be used fully automatically in the whole framework before filtering out such wrong results. On the other hand, we argue that such filtering yields much more reliable metrics for the downstream tasks of mutation classification and survival prediction (as the underlying segmentations do not contain errors). Moreover, this filtering step was performed on the whole-slide level, and as such was extremely fast. This step involves much less human labor than manual selection of ROIs, a step which was involved in previous studies that applied AI to H&E images [(8)](https://www.zotero.org/google-docs/?UGLMXI).

While the number of 26 patients from which we sourced our training patches for ARA-CNN may seem low at first, it is in fact similar to other published datasets. At the same time, the number of extracted training H&E patches is higher compared to previous studies. For instance, Kather *et al.* [(9)](https://www.zotero.org/google-docs/?oRUW5C) extracted 5000 patches from 10 patients, while Spanhol *et al.* [(10)](https://www.zotero.org/google-docs/?0AC35H) extracted 7909 patches from 82 patients. In our case, we extracted 23,199 patches from 26 patients. Furthermore, our set of patients was quite diverse in terms of lung cancer types, which allowed the models trained with *LubLung* to be generalizable at test time.

The *LubLung* dataset was split into training and test sets independent of patients, so e.g. stroma tissue from a single patient could be included as patches in both training and test sets. This fact means that the classification metrics reported for ARA-CNN could be affected by patient-specific effects leaking into the test set. However, all slides from which *LubLung* was sourced were stained consistently, in the same lab, and as such the same tissue type remained consistent across patients as well. While it may be the case that some tissue types differ across patients, this is not a big issue considering the scale of the dataset at 23,199 patches. Moreover, other published datasets [(9,10)](https://www.zotero.org/google-docs/?AJhwIC) of H&E tissue patches were prepared and analyzed in the same way, by assigning patches from different patients into the same class. Note that in order to apply ARA-CNN to the TCGA dataset, we performed normalization to get rid of possible batch effects.

## **References**

[1. Mo J, Hu X, Gu L, Chen B, Khadaroo PA, Shen Z, et al. Smokers or non-smokers: who benefits more from immune checkpoint inhibitors in treatment of malignancies? An up-to-date meta-analysis. World J Surg Oncol. 2020 Jan 20;18:15.](https://www.zotero.org/google-docs/?ZreIxr)

[2. Rączkowska A, Możejko M, Zambonelli J, Szczurek E. ARA: accurate, reliable and active histopathological image classification framework with Bayesian deep learning. Sci Rep. 2019 Oct 4;9(1):14347.](https://www.zotero.org/google-docs/?ZreIxr)

[3. Greenwald NF, Miller G, Moen E, Kong A, Kagel A, Dougherty T, et al. Whole-cell segmentation of tissue images with human-level performance using large-scale data annotation and deep learning. Nat Biotechnol. 2021 Nov 18;1–11.](https://www.zotero.org/google-docs/?ZreIxr)

[4. Jackson HW, Fischer JR, Zanotelli VRT, Ali HR, Mechera R, Soysal SD, et al. The single-cell pathology landscape of breast cancer. Nature. 2020 Feb;578(7796):615–20.](https://www.zotero.org/google-docs/?ZreIxr)

[5. Martin-Gonzalez P, Crispin-Ortuzar M, Markowetz F. Predictive Modelling of Highly Multiplexed Tumour Tissue Images by Graph Neural Networks. In: Reyes M, Henriques Abreu P, Cardoso J, Hajij M, Zamzmi G, Rahul P, et al., editors. Interpretability of Machine Intelligence in Medical Image Computing, and Topological Data Analysis and Its Applications for Medical Data. Cham: Springer International Publishing; 2021. p. 98–107. (Lecture Notes in Computer Science).](https://www.zotero.org/google-docs/?ZreIxr)

[6. Schürch CM, Bhate SS, Barlow GL, Phillips DJ, Noti L, Zlobec I, et al. Coordinated Cellular Neighborhoods Orchestrate Antitumoral Immunity at the Colorectal Cancer Invasive Front. Cell. 2020 Sep 3;182(5):1341-1359.e19.](https://www.zotero.org/google-docs/?ZreIxr)

[7. Khosravi P, Kazemi E, Imielinski M, Elemento O, Hajirasouliha I. Deep Convolutional Neural Networks Enable Discrimination of Heterogeneous Digital Pathology Images. eBioMedicine. 2018 Jan 1;27:317–28.](https://www.zotero.org/google-docs/?ZreIxr)

[8. Mobadersany P, Yousefi S, Amgad M, Gutman DA, Barnholtz-Sloan JS, Vega JEV, et al. Predicting cancer outcomes from histology and genomics using convolutional networks. Med Sci. 2018;10.](https://www.zotero.org/google-docs/?ZreIxr)

[9. Kather JN, Weis CA, Bianconi F, Melchers SM, Schad LR, Gaiser T, et al. Multi-class texture analysis in colorectal cancer histology. Sci Rep. 2016 Jun 16;6(1):27988.](https://www.zotero.org/google-docs/?ZreIxr)

[10. Spanhol FA, Oliveira LS, Petitjean C, Heutte L. A Dataset for Breast Cancer Histopathological Image Classification. IEEE Trans Biomed Eng. 2016 Jul;63(7):1455–62.](https://www.zotero.org/google-docs/?ZreIxr)


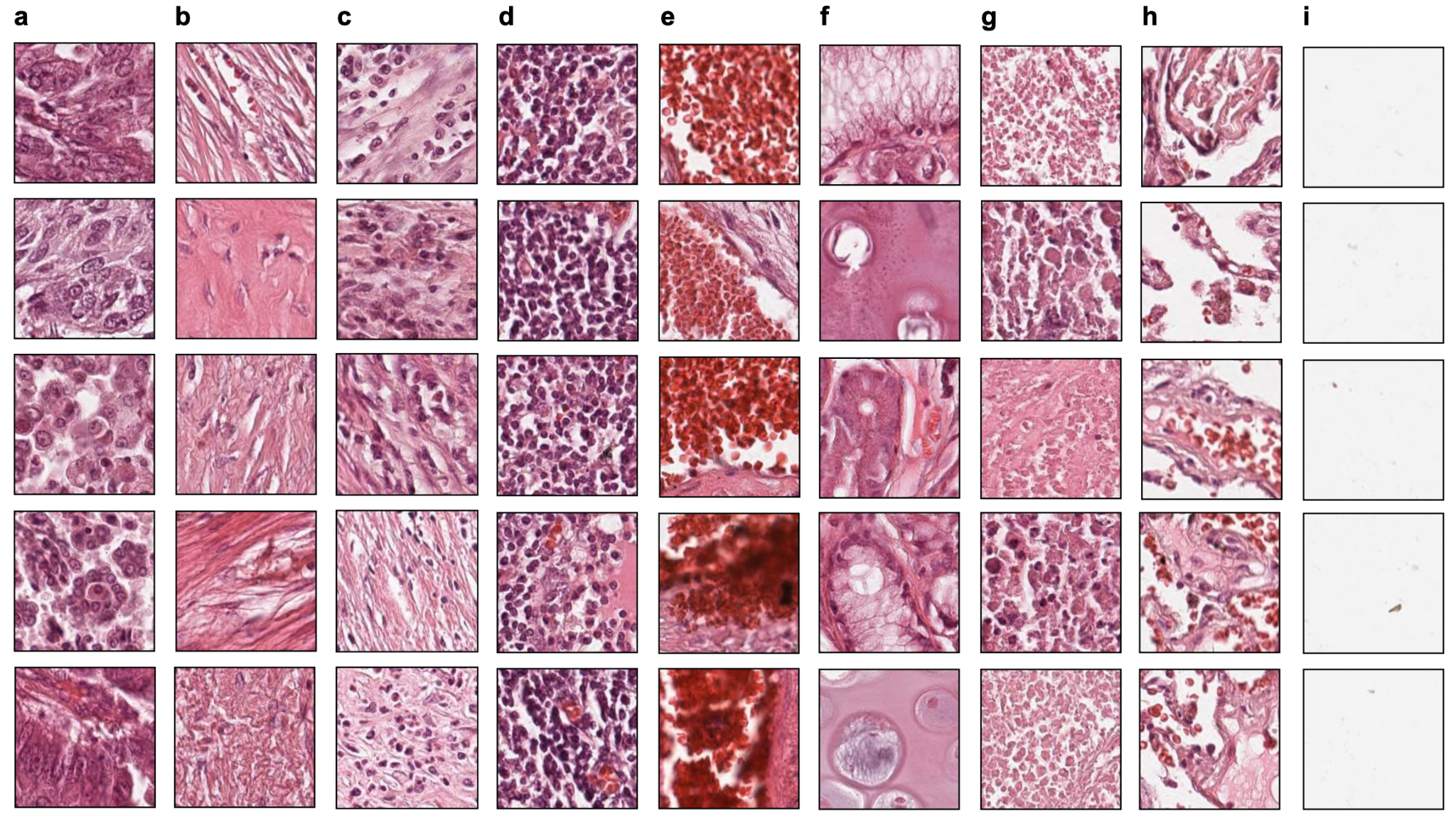


**Supplementary Figure 1.** Example image patches from *LubLung*. **(a)** Tumor class **(b)** Stroma class **(c)** Mixed class **(d)** Immune class **(e)** Vessel class **(f)** Bronchi class **(g)** Necrosis class **(h)** Lung class **(i)** Background class


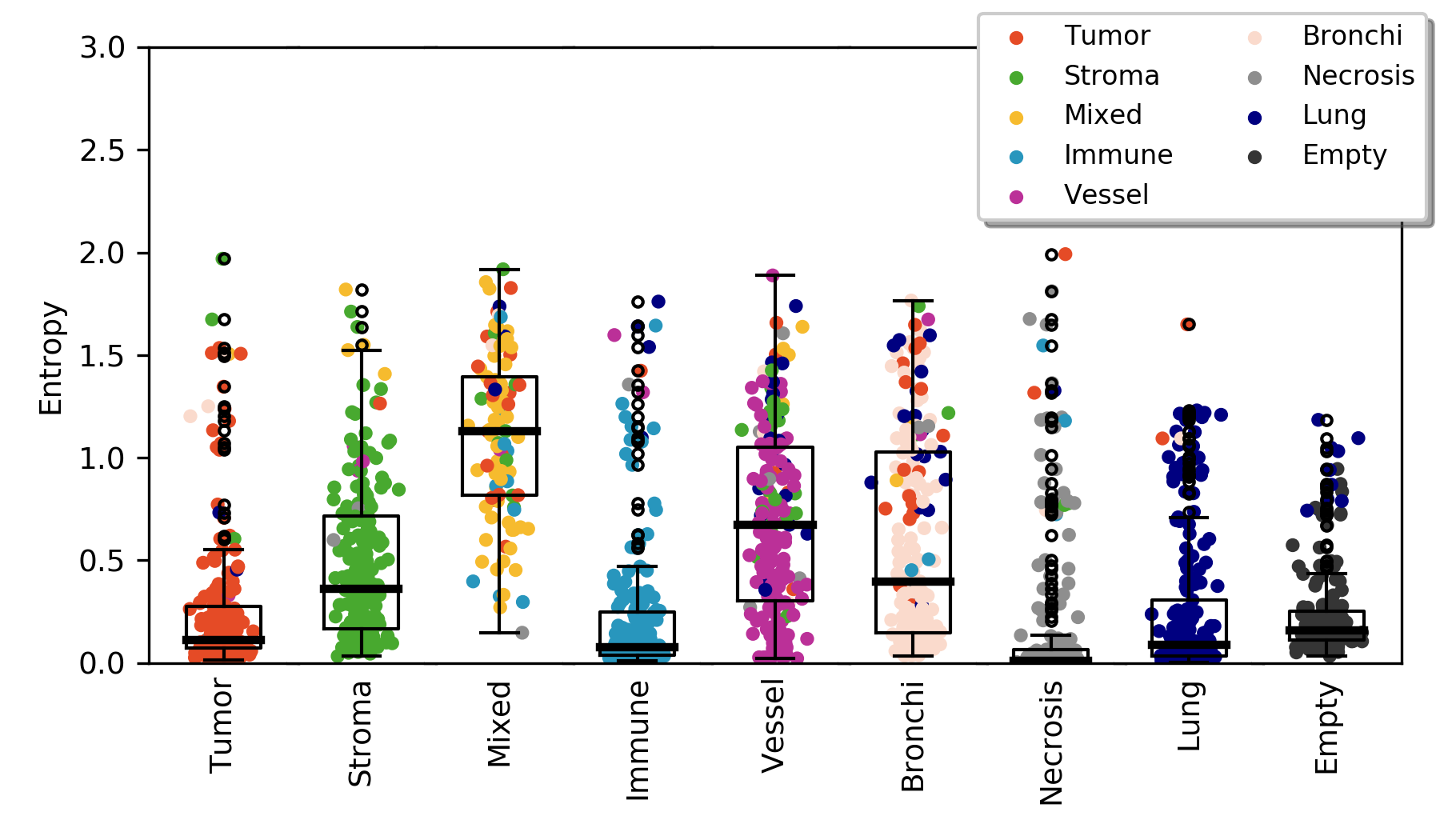


**Supplementary Figure 2.** Per-class uncertainty distribution for the test data points. The test points were aggregated from 10 cross-validation folds resulting from the full *LubLung* dataset of 23,199 tissue patches. The X axis indicates ground truth classes, while the colors of the points encode the model prediction. Based on this result (median entropy for all classes < 1.5), we decided to stop the active learning procedure.


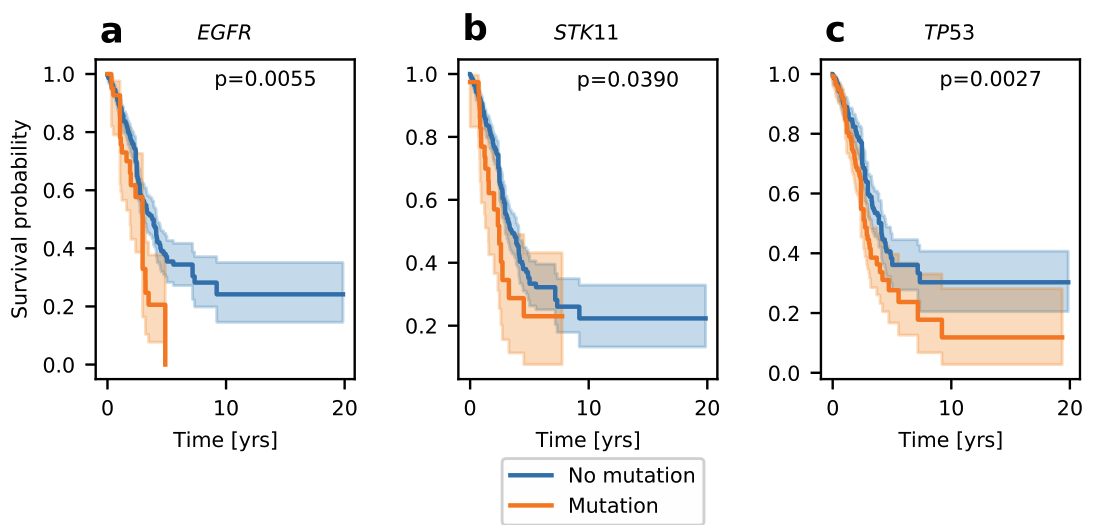


**Supplementary Figure 3.** Kaplan-Meier plots for EGFR, STK11 and TP53 genes, stratified into patients with mutation and patients without mutation. The *p*-values were measured using the log rank test.

**
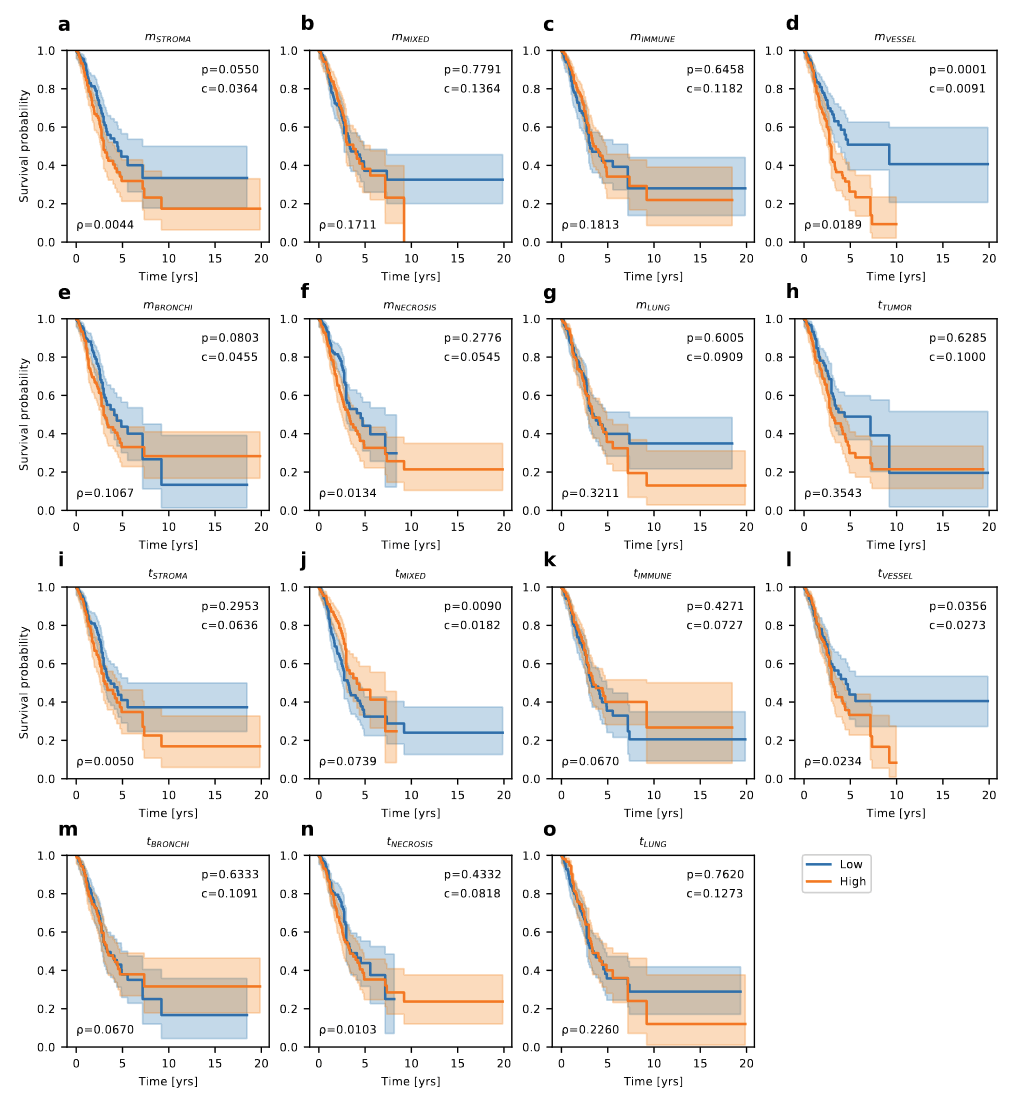
**

**Supplementary Figure 4.** Kaplan-Meier plots for TIP and TMEC features that result in patient stratification into two groups: with high and low values of the feature. The division between high and low value of the feature was based on the median value, indicated by ρ (lower left corner). *p*-values were measured using the log rank test, while critical values *c* were acquired in the Benjamini-Hochberg procedure, with the False Discovery Rate set to 0.1. A result is statistically significant if *p <* 0.05
or *p_r_ < c*, where *p_r_* is a *p*-value ranked in ascending order.


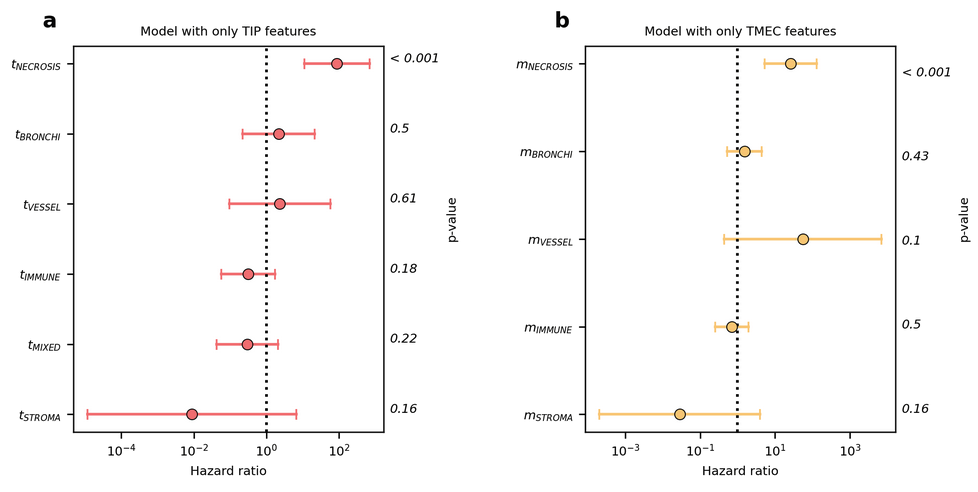


**Supplementary Figure 5.** Hazard ratios for continuous Cox survival models. **(a)** Hazard ratios for a model fitted with TIP features only, where their values were not discretized, but used as-is in continuous Cox regression. The *t_NECROSIS_* feature has a statistically significant negative effect on survival, which is consistent with full analysis presented in the main text. **(b)** Hazard ratios for a model fitted with TMEC features only, where their values were not discretized, but used as-is in continuous Cox regression. The *m_NECROSIS_* feature has a statistically significant negative effect on survival, which is consistent with full analysis presented in the main text.

**
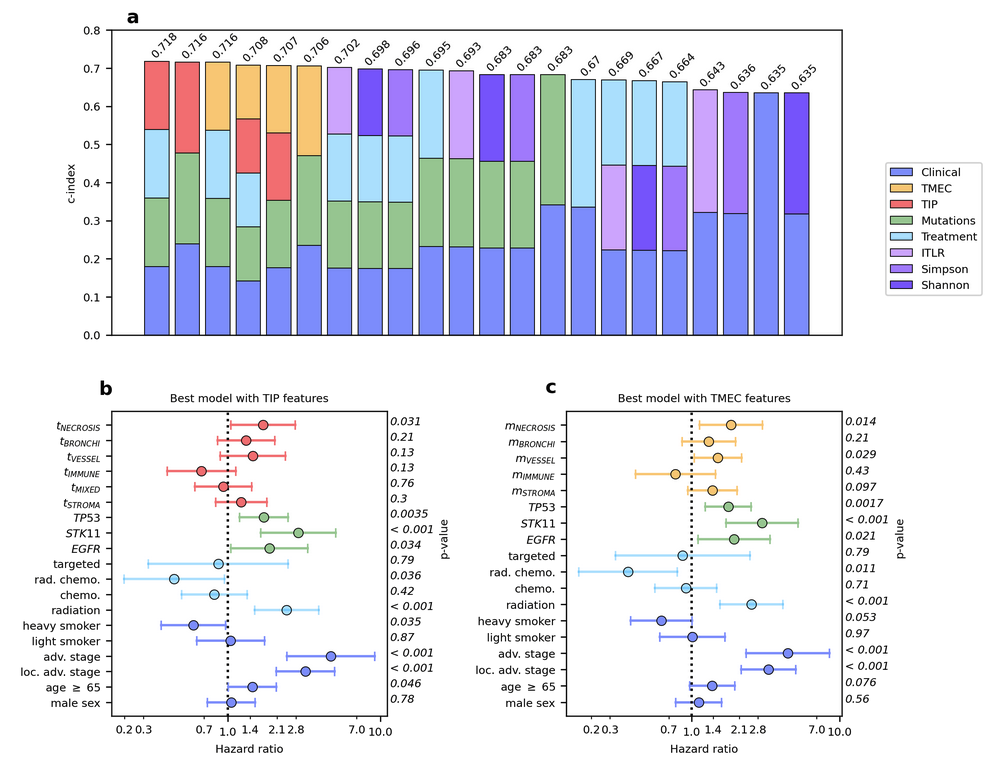
**

**Supplementary Figure 6.** Survival prediction results with treatment information included. **(a)** c-index scores for Cox models from survival prediction experiments performed with different feature sets. The best results were obtained for models with such feature sets that included TIP and TMEC features. Treatment information increased c-index scores when compared to feature sets without it. **(b)** Hazard ratios for the best model that utilized the TIP features. The *t_NECROSIS_* feature has a statistically significant negative effect on survival. **(n)** Hazard ratios for the best model that utilized the TMEC features. The *m_NECROSIS_* feature has a statistically significant negative effect on survival.

**Supplementary Table 1.** Confusion matrix for ARA-CNN trained with patches sized 74 µm extracted from initial tissue annotations. The mean accuracy is 84.35%.

|  | **Tumor [%]** | **Stroma [%]** | **Mixed [%]** | **Immune [%]** | **Vessel [%]** | **Bronchi [%]** | **Necrosis [%]** | **Lung [%]** | **Background [%]** |
| --- | --- | --- | --- | --- | --- | --- | --- | --- | --- |
| **Tumor** | 90.34 | 0.25 | 1.15 | 3.72 | 0.59 | 0.86 | 0.88 | 2.21 | 0.00 |
| **Stroma** | 4.17 | 85.37 | 2.66 | 0.19 | 2.36 | 0.77 | 3.40 | 1.08 | 0.00 |
| **Mixed** | 14.65 | 6.73 | 61.49 | 10.69 | 1.09 | 0.99 | 1.68 | 2.67 | 0.00 |
| **Immune** | 0.59 | 0.32 | 0.48 | 94.89 | 0.59 | 0.16 | 0.65 | 2.15 | 0.16 |
| **Vessel** | 1.21 | 11.66 | 1.41 | 0.25 | 70.70 | 2.36 | 1.51 | 10.80 | 0.10 |
| **Bronchi** | 9.93 | 2.37 | 2.63 | 2.09 | 2.73 | 68.85 | 0.83 | 10.58 | 0.00 |
| **Necrosis** | 0.58 | 0.80 | 0.42 | 0.83 | 1.25 | 0.11 | 95.50 | 0.51 | 0.00 |
| **Lung** | 0.32 | 0.10 | 0.04 | 0.01 | 0.38 | 0.20 | 0.02 | 98.31 | 0.61 |
| **Background** | 0.00 | 0.00 | 0.00 | 0.00 | 0.12 | 0.00 | 0.00 | 3.59 | 96.29 |

**Supplementary Table 2.** Confusion matrix for ARA-CNN trained with patches sized 87 µm extracted from initial tissue annotations. The mean accuracy is 85.21%.

|  | **Tumor [%]** | **Stroma [%]** | **Mixed [%]** | **Immune [%]** | **Vessel [%]** | **Bronchi [%]** | **Necrosis [%]** | **Lung [%]** | **Background [%]** |
| --- | --- | --- | --- | --- | --- | --- | --- | --- | --- |
| **Tumor** | 90.27 | 0.27 | 0.91 | 4.17 | 0.73 | 0.65 | 1.08 | 1.94 | 0.00 |
| **Stroma** | 4.56 | 84.31 | 2.06 | 0.06 | 4.69 | 0.56 | 3.31 | 0.44 | 0.00 |
| **Mixed** | 15.32 | 4.19 | 62.58 | 10.32 | 3.23 | 0.97 | 1.61 | 1.77 | 0.00 |
| **Immune** | 0.58 | 0.08 | 0.58 | 94.79 | 0.74 | 0.08 | 0.58 | 2.15 | 0.41 |
| **Vessel** | 0.80 | 9.73 | 1.47 | 0.20 | 75.07 | 1.60 | 1.67 | 9.40 | 0.07 |
| **Bronchi** | 8.32 | 1.42 | 2.84 | 2.53 | 2.68 | 69.21 | 0.63 | 12.32 | 0.05 |
| **Necrosis** | 0.79 | 0.52 | 0.23 | 0.47 | 0.77 | 0.09 | 96.82 | 0.32 | 0.00 |
| **Lung** | 0.53 | 0.88 | 0.08 | 0.13 | 1.19 | 0.45 | 0.20 | 95.78 | 0.76 |
| **Background** | 0.00 | 0.00 | 0.00 | 0.00 | 0.00 | 0.00 | 0.00 | 1.95 | 98.05 |

**Supplementary Table 3.** Confusion matrix for ARA-CNN trained with patches sized 100 µm extracted from initial tissue annotations. The mean accuracy is 84.35%.

|  | **Tumor [%]** | **Stroma [%]** | **Mixed [%]** | **Immune [%]** | **Vessel [%]** | **Bronchi [%]** | **Necrosis [%]** | **Lung [%]** | **Background [%]** |
| --- | --- | --- | --- | --- | --- | --- | --- | --- | --- |
| **Tumor** | 88.22 | 0.44 | 1.63 | 3.89 | 0.59 | 1.33 | 1.56 | 2.33 | 0.00 |
| **Stroma** | 6.58 | 80.17 | 2.05 | 0.34 | 3.68 | 1.37 | 4.87 | 0.94 | 0.00 |
| **Mixed** | 12.83 | 4.78 | 60.65 | 11.52 | 1.74 | 1.74 | 3.48 | 3.26 | 0.00 |
| **Immune** | 0.88 | 0.25 | 0.63 | 95.38 | 1.00 | 0.00 | 0.50 | 1.38 | 0.00 |
| **Vessel** | 1.43 | 9.81 | 0.76 | 0.29 | 74.19 | 1.62 | 2.57 | 9.14 | 0.19 |
| **Bronchi** | 9.04 | 1.76 | 1.76 | 2.35 | 2.65 | 67.87 | 0.96 | 13.60 | 0.00 |
| **Necrosis** | 0.60 | 0.40 | 0.23 | 0.63 | 0.80 | 0.13 | 97.00 | 0.20 | 0.00 |
| **Lung** | 0.36 | 0.59 | 0.02 | 0.13 | 0.43 | 0.27 | 0.34 | 97.15 | 0.70 |
| **Background** | 0.00 | 0.00 | 0.00 | 0.00 | 0.00 | 0.00 | 0.00 | 1.47 | 98.53 |
